# Intracellular activity of tedizolid phosphate and ACH-702 versus *Mycobacterium tuberculosis* infected macrophages

**DOI:** 10.1186/1476-0711-13-13

**Published:** 2014-04-04

**Authors:** Carmen A Molina-Torres, Alejandra Barba-Marines, Orestes Valles-Guerra, Jorge Ocampo-Candiani, Norma Cavazos-Rocha, Michael J Pucci, Jorge Castro-Garza, Lucio Vera-Cabrera

**Affiliations:** 1 Servicio de Dermatología, Hopital Universitario, UANL, C.P, Monterrey, NL 64460, Mexico; 2 Achillion Pharmaceuticals, Inc., New Haven, CT, USA; 3 División de Biología Celular y Molecular, Centro de Investigación Biomédica del Noreste, IMSS, Monterrey, Mexico; 4 Departamento de Quimica Analítica, Facultad de Medicina, UANL, Monterrey, Mexico

**Keywords:** ACH-702, Tuberculosis, Oxazolidinones

## Abstract

**Background:**

Due to the emergency of multidrug-resistant strains of *Mycobacterium tuberculosis*, is necessary the evaluation of new compounds.

**Findings:**

Tedizolid, a novel oxazolidinone, and ACH-702, a new isothiazoloquinolone, were tested against *M. tuberculosis* infected THP-1 macrophages. These two compounds significantly decreased the number of intracellular mycobacteria at 0.25X, 1X, 4X and 16X the MIC value. The drugs were tested either in nanoparticules or in free solution.

**Conclusion:**

Tedizolid and ACH-702 have a good intracellular killing activity comparable to that of rifampin or moxifloxacin.

## Background

Tuberculosis (TB) is a chronic respiratory disease that in most cases is treatable and curable. Emergence of multidrug resistant TB strains (MDR), defined as those resistant to at least rifampin and isoniazid, has been a public health threat around the world. Recently, severe forms of drug resistance, such as extensively drug-resistant (XDR) TB, have been described. According to the WHO, there were about 310,000 cases of MDR-TB among notified patients with pulmonary TB in the world in 2011. Almost 60% of these cases were reported in India, China and the Russian Federation. It is estimated that about 9% of MDR-TB cases also had XDR-TB [1]. Given this increase in global resistance, new compounds or new therapeutic schemes are urgently needed.

Tedizolid phosphate (Trius Therapeutic Inc, San Diego California) (formerly known as DA-7218, Dong-A Pharmaceutical Company, Ltd., Yongin, Korea) is a novel, potent oxazolidinone pro-drug which is rapidly converted *in vivo* to active tedizolid with antimicrobial effects. Tedizolid interacts with the bacterial 23S ribosome initiation complex to inhibit translation, and is active against all clinically relevant gram-positive pathogens, including linezolid-resistant *Staphylococcus aureus*[2]. At present, this drug is in Phase III clinical trials for treating acute bacterial skin and skin structure infections with good results, similar to those obtained with linezolid [3].

ACH-702 is a new isothiazoloquinolone that inhibits bacterial DNA replication through inhibition of DNA gyrase and topoisomerase IV. It has potent antibacterial activity against a number of medically relevant bacteria, including drug-resistant strains such as methicillin-resistant *S. aureus* (MRSA) and vancomycin-resistant enterococci, (VRE) [4-6]. Those two drugs have shown excellent *in vitro* activity against susceptible and multidrug resistant *M. tuberculosis* clinical isolates [7,8]. Drugs in tuberculosis have to taken for months. Tedizolid belongs to the same family of oxazolidinones as linezolid. Clinical experience with the latter has shown the appearing of side effects, particularly mielossupression and peripheral neuropathy after several months of application [9]. Quinolones, particularly gatifloxacin, can produce disglicemias when is applied chronically [10]. Given these data we wanted to evaluate the possibility to use tedizolid or ACH-702 them in particulate form, which would allow the use of higher doses with less toxicity.

Nanoparticles have been proposed as an improved system to carry and deliver drugs to a target organ and have considerable potential for TB treatment [11]. Nanoparticles show advantages as drug carriers because of high stability, capability to incorporate either hydrophilic or hydrophobic compounds, and administration route flexibility [11]. Hence, the main aim of this paper was to determine the intracellular activity against M. tuberculosis of those two recently developed drugs: tedizolid and ACH7-702 in two different forms: dissolved in an adequate solvent and encapsulated in the synthetic, biodegradable/biocompatible polymer; the poly-lactide-co-glycolide (PLG), which has been approved by the US FDA for human use [12].

## Methods

### Culture

*M. tuberculosis* H37Rv (ATCC 27294) strain was grown on Lowenstein-Jensen media and then inoculated to liquid Middlebrook 7H9 medium for 7 days at 37°C. From this culture, CFUs were quantitated by plating in Midlebrook 7H10 agar.

### Broth microdilution assay

Tedizolid Phosphate was donated by Sung-Hak Choi from Dong-A Pharmaceutical Company, Ltd., Yongin, Korea, while ACH-702 was obtained from Achillion Pharmaceuticals, Inc., New Haven. Stock solutions of 1 mg/ml for moxifloxacin and tedizolid were dissolved in water, ACH-702 was dissolved in dimethyl sulfoxide and rifampin was dissolved in 95% ethanol. The Minimal Inhibitory Concentration (MIC) for each drug was determined using the broth microdilution method with Alamar Blue [13]. In brief, mycobacterial suspensions were prepared in 0.04% (vol/vol) Tween 80–0.2% bovine serum albumin so their turbidities equaled a McFarland turbidity standard of 1. Suspensions were further diluted 1:25 in 7H9GC broth. The rest of the technique was performed as published before [13]. The MIC was defined as the lowest drug concentration which prevented a color change of blue to pink. MICs determined of each drug were: rifampin: 0.125 μg/ml, moxifloxacin: 0.125 μg/ml, ACH-702: 0.063 μg/ml and Tedizolid: 1.0 μg/ml.

### Preparation of PLG-nanoparticles

Drug-loaded PLG-nanoparticles were prepared by the multiple emulsion and solvent evaporation technique described previously [14-17]. Briefly, 10 mg of drug and 10 mg of PLG were dissolved in distilled water, and then added to dichloromethane (DCM) [water/DCM 1:10 (v/v)] to a final volume of 10 ml. The mixture was sonicated for 1 min to form the primary emulsion, which was poured into 1% (w/v) aqueous polyvinyl alcohol and re-sonicated for 3 min. The secondary emulsion formed was stirred overnight and centrifuged at room temperature (8,000–10,000 rpm for 15 min) to remove DCM and to harvest the nanoparticles, which were washed three times with distilled water and finally resuspended with 5 ml of water.

### Quantification of PLG-nanoparticles

In order to quantify the encapsulated drug, an aliquot of 100 μl was diluted in 900 μl of 5% (w/v) SDS in 0.1 M NaOH (lysis reagent) for 30 min at 50°C to release the encapsulated drugs. The drugs were quantitated using a Beckman DU-7500 UV-Visible, Scanning Spectrophotometer (Brea, California, USA), using 486 nm as the detection wavelength for rifampicin and 286 nm as the detection wavelength for the rest of antimicrobials using lysis reagent as blank and the standards as controls. Antimicrobials in nanoparticle form and free in solution were prepared from the stock solution to final concentrations of 0.25X, 1X, 4X and 16X of the MIC previously determined.

### Drug cytotoxicity assay

In order to test the effect of drugs over THP-1 cells an assay for cytotoxicity was carried on. THP-1 cells were transformed to macrophages as described previously [18] and then incubated at concentrations 16X the MIC for *M. tuberculosis* H37Rv (ACH-702: 2 μg/ml, Tedizolid: 8 μg/ml, Rifampicin: 15 μg/ml and Moxifloxacin: 8 μg/ml). After 12 h exposure (time used in posterior experiments) viability was assessed using the crystal violet technique [18,19]. In all cases cell viability after drug treatment was greater than 98%.

### Intracellular antibacterial assays

Intracellular antibacterial assays were done according to the technique previously reported [20] as follows: before the beginning of the experiment, bacteria were thawed at 37°C and adjusted to 4 × 10^5^ bacteria per milliliter with RPMI-1640 medium; the monolayers were infected at a multiplicity of infection of 1:10 (cells:bacilli) and incubated for 6 h at 37ºC in a humid atmosphere with 5% CO_2_. In order to remove the extracellular bacteria, cultures were washed twice with pre-warmed PBS at 37ºC and then 1 ml of amikacin (200 μg/ml) was added to each well followed by incubation under the same conditions as above for 2 h. The wells were washed twice with PBS and 1 ml of RPMI-1640 containing the drug in solution or encapsulated to be tested was added, and the plate was incubated for 12 h. After the pulse of antimicrobials, they were removed from the wells and 1 ml RPMI-1640 was added. Three wells were used to quantitate the initial CFUs by agar plating and the rest of the wells incubated for an additional 60 h. At the end of the incubation time, bacterial counts were determined from each well by agar plating as before. Plates were incubated at 37ºC for 7 to 10 days until colonies were visible. Data were evaluated with ANOVA variance for multiple comparisons test.

## Results and discussion

**Figure 1:**
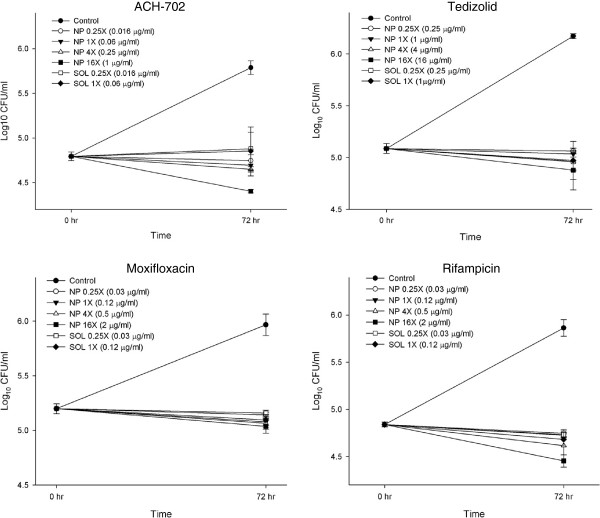
**THP-1 cells infected with *Mycobacterium tuberculosis *H37Rv to a M.O.I.: 1:10 (cells:bacilli) were subjected to several concentrations of drugs in solution (0.25X and 1X MICs) and nanoparticles (0.25X, 1X, 4X and 16X MICs) in triplicate as described.** After 72 h post infection, cells were lysed and plated on 7H10 to determine CFU/ml. Plots detail the bactericidal activity of ACH-702, Tedizolid, Moxifloxacin and Rifampicin. Values represent means ±1 standard error of the means.

The intracellular antimicrobial activity of tedizolid and ACH-702 against *M. tuberculosis* was assessed using the THP-1 monocytic cell line since it has been a reliable model to examine intracellular infection with *M. tuberculosis*[20]. As shown in Figure 1, tedizolid and ACH-702 each dramatically decreased the number of intracellular mycobacteria 72 h after the infection compared with the control when they were exposed to concentrations 0.25 X, 1 X, 4X and 16X MICs for the nanoparticulated form (NP) and 0.25X and 1X MICs for the free solution (SOL) (p < 0.0001). ACH -702 treatment led to a reduction of 1.4 LOG_10_ UFC/ml and tedizolid decreased bacterial counts 1.3 LOG. This activity can be comparable to that elicited by two antibiotics with proven activity against tuberculosis, moxifloxacin (reduced 1 LOG) and rifampicin (reduced 1.4 LOG). Rifampicin is still used as part of the first line drug regimen due to sterilizing activity against non-dividing bacteria and moxifloxacin is one of the drugs being analyzed as part of the second line TB drugs because of its low CMI and a rapid sterilization activity on mycobacteria [21,22]. Since we previously eliminated extracellular bacteria with amikacin in our assay, the antibacterial activity of Tedizolid and ACH-702 was intracellular. This result is promising on the possible use of these drugs during active infection.

A strategy to specifically deliver chemotherapeutic compounds formulated in nanoparticles to target disease sites has been discussed over the last two decades [23]. Several drug carriers made of biocompatible and biodegradable materials have been used including polymeric nanoparticles, where the drug is attached, entrapped or encapsulated in polymeric core and, depending upon the method of preparation, they are designated as nanoparticles, nanospheres or nanocapsules [24].

Under nanoparticle format isoniazid, rifampicin, pyrazinamide, moxifloxacin and econazole have been prepared and administrated by gavage, subcutaneous and pulmonary route to murine and guinea pig models of tuberculosis [23]. In all the studies made so far, nanoparticles produced more prolonged serum levels of the drug than its free version, and also less doses of nanoparticle form were sufficient to achieve bacterial clearance and in some cases resulted in complete sterilization [23]. In our study, there was not a significant difference in the antimicrobial activity between drug in solution and nanoparticles probably due to the high efficacy exhibited by drugs at low concentrations.

## Conclusions

*Mycobacterium tuberculosis* is a bacteria living preferentially inside the cells. One step ahead in research of anti-TB drugs is to evaluate their intracellular activity against the bacilli. In this paper we have described a suitable assay in THP-1 macrophages to study anti-tuberculosis compounds on intracellular bacteria. We found that both drugs tedizolid and ACH-702 had a comparable activity to that elicited by rifampin and moxifloxacin. However we did not find differences if the compounds were in solution or encapsulated in PLG. Further studies on these drugs should consider using an *in vivo* animal model to explore their possibility as anti-tuberculosis therapy.

## Abbreviations

TB: Tuberculosis; MDR: Multi-drug resistance phenotypes; XDR: Extensively drug resistant phenotypes; MRSA: *Staphylococcus aureus* methicillin resistant; VRE: Vancomicin resistant enterococci; PLG: Poly-lactide-co-glycolide; U.S. FDA: United States Food and Drug Administration; CFUs: Colony forming units; MIC: Minimal inhibitory concentration; DCM: Dichloromethane; LOG: Logarithm.

## Competing interests

The authors declare that they have no competing interests.

## Authors’ contributions

CAMT and LVC participated in the design of the study and wrote the manuscript. OVG and ABM carried out the experimental studies. NCR quantitated the drugs. JCG and MJP participated in critical revision of manuscript and data content. JOC helped in drafting the manuscript. All authors read and approved the final manuscript.
